# Development of Genetic Markers in *Eucalyptus* Species by Target Enrichment and Exome Sequencing

**DOI:** 10.1371/journal.pone.0116528

**Published:** 2015-01-20

**Authors:** Modhumita Ghosh Dasgupta, Veeramuthu Dharanishanthi, Ishangi Agarwal, Konstantin V. Krutovsky

**Affiliations:** 1 Division of Plant Biotechnology, Institute of Forest Genetics and Tree Breeding, P.B. No. 1061, R.S. Puram, Coimbatore–641002, India; 2 Genotypic Technology Private Limited, #2/13, Balaji Complex, Poojari Layout, 80, Feet Road, R. M. V. 2nd Stage, Bangalore-560094, India; 3 Department of Forest Genetics and Forest Tree Breeding, Büsgen Institute, Georg August University of Göttingen, Büsgenweg 2, D-37077 Göttingen, Germany; 4 Department of Ecosystem Science and Management, Texas A&M University, 2138 TAMU, College Station, TX 77843-2138, United States of America; 5 N.I. Vavilov Institute of General Genetics, Russian Academy of Sciences, Moscow 119333, Russia; 6 Genome Research and Education Center, Siberian Federal University, 50a/2 Akademgorodok, Krasnoyarsk 660036, Russia; National Institute of Plant Genome Research (NIPGR), INDIA

## Abstract

The advent of next-generation sequencing has facilitated large-scale discovery, validation and assessment of genetic markers for high density genotyping. The present study was undertaken to identify markers in genes supposedly related to wood property traits in three *Eucalyptus* species. Ninety four genes involved in xylogenesis were selected for hybridization probe based nuclear genomic DNA target enrichment and exome sequencing. Genomic DNA was isolated from the leaf tissues and used for on-array probe hybridization followed by Illumina sequencing. The raw sequence reads were trimmed and high-quality reads were mapped to the *E. grandis* reference sequence and the presence of single nucleotide variants (SNVs) and insertions/ deletions (InDels) were identified across the three species. The average read coverage was 216X and a total of 2294 SNVs and 479 InDels were discovered in *E. camaldulensis*, 2383 SNVs and 518 InDels in *E. tereticornis*, and 1228 SNVs and 409 InDels in *E. grandis*. Additionally, SNV calling and InDel detection were conducted in pair-wise comparisons of *E. tereticornis* vs. *E. grandis*, *E. camaldulensis* vs. *E. tereticornis* and *E. camaldulensis* vs. *E. grandis*. This study presents an efficient and high throughput method on development of genetic markers for family– based QTL and association analysis in *Eucalyptus*.

## Introduction

The genus *Eucalyptus* belongs to family Myrtaceae and consists of over 700 species [1] that occupy a broad range of environmental conditions. Most of the species are native to Australia and have been introduced to India, France, Chile, Brazil, South Africa and Portugal in the first quarter of 1800s [2]. It is one of the most widely planted hardwood crop in the world because of its superior growth, adaptability and wood properties and occupies 20.07 M hectares globally. India ranks second in area under *Eucalyptus* plantation (3.943 M ha) after Brazil (4.259 M ha) [3]. In tropical and subtropical regions, *E. grandis, E. urophylla* and their hybrids are highly preferred for pulp production and solid wood, while *E. globulus* is favored in the temperate regions [4]. Six species including *E. camaldulensis, E. grandis, E. globulus, E. pellita, E. tereticornis* and *E. urophylla* are reported to be suitable for Indian agro-climatic conditions and widely planted in the subcontinent [5–6].

*Eucalyptus* is a potential out-crosser and due to unlimited free natural hybridizations, the populations are highly heterozygous. Hence, extensive studies were conducted to determine genetic diversity at species and population levels using different marker systems [7–16].

Linkage maps in different species of Eucalypts have been widely reported [17–21]. QTL mapping in this genus has been conducted tagging important traits like wood properties, vegetative propagation, response to biotic and abiotic stress, juvenile traits, stem growth, water stress tolerance and frost tolerance [22–27]. QTL studies in *Eucalyptus* species was recently reviewed in detail by Grattapaglia *et al*. [28]. Population based association studies were reported for *E. nitens* and *E. globulus* targeting wood property traits [29–31]. Recently, the first experimental study of Genomic Selection was reported by Resende and co workers [32] in two *Eucalyptus* populations for growth and wood property traits.

The genomic data in *Eucalyptus* species are well-documented and available in public databases, private collections and consortia as EST resources [33–34] and transcriptome resources [16, 35–42]. Several dedicated databases are available for *Eucalyptus* genome research, such as EUCANEXT, *Eucalyptus*DB, Eucspresso [38], EUCATOUL, EUCAWOOD [33], EucaCold [34], EucGenIE [43] and Phytozome10.

Subsequently, the *Eucalyptus* genome sequencing project was initiated independently for *E. grandis* at the US Department of Energy Joint Genome Institute, USA and *E. camaldulensis* at Kazusa DNA Research Institute in Japan. Recently, the complete genome sequence of *E. grandis* (‘BRASUZ1’) was published [44] and the assembled non-redundant chromosome-scale reference (v1.0) was released with 640 Mb (94%) genome coverage organized into 11 pseudomolecules. It was also reported that 34% of the protein-coding genes occur as tandem duplication and 84% share similarity to rosid lineages.

The draft genome sequence of *E. camaldulensis* sequenced in Japan had a total length of 655,922,307 bp of non-redundant genomic sequences consisting of 81,246 scaffolds and 121,194 singlets. These sequences accounted for approximately 92% of the gene-containing regions. A total of 77,121 complete and partial structures of protein-encoding genes were annotated [45]. The database containing the draft sequence can be accessed at http://www.kazusa.or.jp/eucaly.

In the last decades several generic DNA markers have been employed for molecular breeding. These markers are usually effective but their development is labor-intensive and time consuming. However, with the advent of ‘next generation’ sequencing technologies, a paradigm shift has occurred in DNA sequencing approach, resulting in high throughput and cost effective sequencing methods [46–47]. Nevertheless, sequencing of large number of genomes is still not feasible due to the substantial cost, time, management and storage of the enormous informatics data. Hence, considerable effort has been directed towards sequencing of genome sub-regions by ‘target enrichment’ methods. Re-sequencing of these enriched genomic regions is time and cost effective and the data analysis is less complex [48].

In the present study, we conducted target enrichment of exomes for 94 genes involved in xylogenesis and re-sequenced them in three *Eucalyptus* species, which were used in developing mapping pedigrees. Presence of SNVs and InDels across different species in pair-wise comparisons and in comparison to the *E. grandis* reference genome was documented. This study presents an efficient and high throughput method on development of genetic markers for family – based QTL and Association analysis in *Eucalyptus*.

## Materials and Methods

### Plant Material and DNA Isolation

Three genotypes from *Eucalyptus camaldulensis, E. tereticornis* and *E. grandis* were selected for target enrichment. *E. camaldulensis* (Ec111) belonging to Kennedy River Provenance from Queensland, Australia is a selection from the Provenance Resource Stand, Pudukkotai, Tamil Nadu, India while *E. tereticornis* (Et86) is a selection from Seed Production Area, Pudukkotai, Tamil Nadu, India. *E. grandis* (Eg9) is a selection from the Lorne provenance trial at Hossammund, Ootacamund, Tamil Nadu, India. These genotypes were used as parents for development of mapping populations targeting wood property traits.

The leaf tissues from the three genotypes were harvested and immediately frozen at −80^°^C. Genomic DNA was isolated from the leaf tissues using the GenElute Plant Genomic DNA isolation kit (Sigma Aldrich, USA) and quantified using NanoDrop ND1000 spectrophotometer (Thermo Scientific, USA).

### Selection of Genes and Probe Design for Sequence Capture Array

Genes involved in different steps of secondary xylem formation including cell division, cell expansion, cell wall thickening, cell wall proteins, lignin biosynthesis and programmed cell death in *Arabidopsis, Populus, Zinnia* and *Eucalyptus* spp. were short-listed from literature and 94 genes were selected for target enrichment and re-sequencing. Their respective gene orthologs were downloaded from *E. grandis* genome database hosted by Phytozome portal (http://www.phytozome.net/cgi-bin/gbrowse/ *Eucalyptus*). The sequences were functionally annotated and their position in chromosome, protein domains, biological pathways and gene ontology were defined based on the recent assembly of *E. grandis* using Phytozome v10 [44].

Hundred and twenty bp long hybridization probes (“baits”) were designed with 1bp tilling using SureSelect eArray software (Agilent Technologies, Santa Clara, California, USA) targeting exons and UTRs in 94 genes. A total of 169,700 baits were designed to capture the exons and UTRs in the three species. Using this design, a customized array was synthesized at Agilent Technologies.

### Library Preparation, Target Enrichment and Validation

Ten micrograms of DNA from each sample in 100 μl of nuclease free water were sonicated to fragment DNA to size range of 100 to 500 bp. The size distribution was checked on the Agilent 2100 Bioanalyzer, and the DNA was cleaned using the Agencourt AMPure XP SPRI beads (Beckman Coulter, Australia). The libraries for each sample were prepared using the Illumina TruSeq DNA Sample Preparation Kit (Illumina Inc., San Diego, CA, USA). The sheared DNA was subjected to a series of enzymatic reactions that repair frayed ends, phosphorylated the fragments, added a single nucleotide overhang to code the libraries and ligated adaptors using manufacturer’s protocol for the Illumina TruSeq DNA sample preparation kit. Subsequently, PCR enrichment (10 cycles) was performed to amplify the library. The three barcoded libraries were pooled in equimolar amounts and approximately 20mg of DNA was hybridized on the Agilent 244Kmicroarray (AMADID: EA560-037734) following manufacturers’ protocol. The hybridization was carried out at 65^°^C for 65 hrs as described by Hodges *et al*. [49]. After standard washing procedures, DNA was eluted in nuclease free water by incubating the array at 95^°^C for 10 min. The captured library was PCR amplified for 18 cycles and purified using the Agencourt AMPure XP SPRI beads (Beckman Coulter, Australia).

The enriched library was quantified using a NanoDrop Spectrophotometer and the quality was checked on the Agilent High Sensitivity Bioanalyzer Chip. RT-qPCR was conducted on pre- and post-captured library using primer pairs designed for the target (*EtCesA1, EtCesA2* and *EtCesA5*) and non-target (*EteIF4* and *EtH2B*) genes (S1 Table) to confirm enrichment of the targeted regions. The qRT-PCR data was analyzed using the ΔΔCT method described by Livak and Schmittgen [50].

### Sequencing and Analysis

The three pooled barcoded libraries were subjected to cluster generation and 2 × 100bp paired end sequencing was conducted using the Illumina GAII Analyzer. High Quality (HQ) reads were filtered from raw data using SeqQC_V2.2 (a proprietary QC tool of Genotypic Technologies Ltd., Bangalore, India) with cutoff Phred quality scores (Q) of 20 (the probability of 1 in 100 bases sequenced may be due to an error). Further, the quality passed sequencing reads were trimmed for Adapter, B Block and low quality end sequences with 50bp cut off using Raw Data Processing Script. The trimmed reads were aligned (gapped alignment) to the *E. grandis* reference sequence using bowtie 2-2.0.0-beta5 [51] with affine read gap penalty and affine reference gap penalty of 5 for gap open and 3 for gap extension. The un-gapped alignment was done using bowtie version 0.12.7 [52]. The variations across the aligned sequences were taken into account from both gapped and un-gapped alignments to overcome the possibilities of false variations induced by allowing gaps. Variations reported in both alignments are expected to be of higher confidence. SNV calling and InDel detection was done using SAMtools version 0.1.7a (http://samtools.sourceforge.net) with default parameters [53]. The cut off thresholds of 3 and 10 were set for the minimum number of reads showing variation and for the minimum RMS mapping quality for SNVs, respectively. The same tool was used to generate the consensus sequence of the aligned reads, while multiple alignments were done using ClustalW version 2.0.12. Pair wise comparison of the sequence data for the three species was conducted to identify SNVs and InDels based on their positions using R Bioconductor code. The ambiguous SNVs generated due to genetic divergence of the three species were not considered for analysis.

## Results and Discussion

### Selection of Candidate Genes

Ninety four xylogenesis-related genes involved in different stages of wood formation including biosynthesis of lignin, cellulose, pectin, monoterpene, xyloglucan, cell wall related genes, genes involved in carbohydrate metabolism, programmed cell death, phyto-hormone signaling, transcription factors and regulatory proteins were selected for the present study (Table 1). The position of the genes in chromosomes and their biological functions in respect to the *E. grandis* reference genome are presented in S2 Table. As many as 14 genes were localized on chromosome 7, while only 4 genes localized on chromosome 8. Two genes, monoterpene glucosyl transferase and IAA binding domain were not assigned to any chromosome.

**Table 1:** Genes selected for target enrichment.

**S.No.**	**Gene ID**	**Gene Product**	**CDS length (bp)**	**Transcript length (bp)**	**Biological Function**	**Xylogenesis-related function**
1	*4CL*	4-Coumarate CoA Ligase	1635	2068	Provides activated thioester substrates for phenylpropanoid natural product biosynthesis	Lignin biosynthesis
2	*ACO1*	Aminocyclopropane-1-carboxylate oxidase	963	1296	Conversion of ACC to the gaseous hormone ethylene	Ethylene signaling
3	*ADH*	Alcohol dehydrogenase	1896	2173	Catalyzes the NAD+−dependent oxidation of alcohols	Alcohol fermentation
4	*AP2L*	APETALA TF	738	1052	Key regulators of several developmental processes like floral development	Regulate secondary wall biosynthesis
5	*ARF1*	Auxin response factor	2364	3228	Transcription factors that bind to TGTCTC auxin response elements in promoters of early auxin response genes	Auxin Signaling. Key regulator of cambium activity and wood formation
6	*ARF2*	Auxin response factor	2520	3949	TF that bind to auxin response elements in promoters of early auxin response genes	Auxin signaling
7	*ASP*	Aspartyl protease	1218	1218	Proteolytic enzyme	Programmed cell death
8	*BFN1*	S1/P1 Nuclease induced during senescence	912	1205	Degradation of RNA and single-stranded DNA	Programmed cell death
9	*BP*	KNAT Knotted like Homeobox TF	1164	1820	Regulates secondary cell wall biosynthesis	Secondary cell wall biogenesis
10	*bZIP*	Basic region/leucine zipper motif TF	858	858	Regulate pathogen defence, light and stress signalling	Stem development and xylem fibre differentiation
11	*C3H*	Coumarate 3- hydroxylase	1530	2048	Hydroxylation of p-coumarate to form caffeate	Lignin biosynthesis
12	*C4H*	Cinnamate 4- hydroxylase	1518	1928	Catalyzes the conversion of cinnamate into 4-hydroxy-cinnamate	Lignin biosynthesis
13	*CAD1*	Cinnamyl alcohol dehydrogenase	1158	1583	Conversion of coniferaldehyde to coniferyl alcohol	Lignin biosynthesis
14	*CAld5H*	Coniferaldehyde 5- hydroxylase	1590	2026	Conversion of confieraldehyde to hydroxyconiferaldehyde	Lignin biosynthesis
15	*CCAAT*	CBF TF	453	453	Cis acting element with diverse functions	
16	*CCoAOMT1*	Caffeoyl-CoA-O-methyl transferase 1	741	1266	Methylation of the 3-hydroxyl group of caffeoyl CoA	Lignin biosynthesis
17	*CesA1*	Cellulose synthase 1	2937	2937	Cellulose deposition in developing secondary xylem	Cellulose biosynthesis
18	*CesA2*	Cellulose synthase 2	2775	3136	Cellulose deposition in developing secondary xylem	Cellulose biosynthesis
19	*CesA3*	Cellulose synthase 3	3126	3126	Cellulose deposition in developing secondary xylem	Cellulose biosynthesis
20	*CesA4*	Cellulose synthase 4	3243	3951	Cellulose deposition in primary cell wall	Primary cell wall formation
21	*CesA5*	Cellulose synthase 5	1710	2136	Cellulose deposition in primary cell wall	Primary cell wall formation
22	*CesA6*	Cellulose synthase 6	2691	3208	Cellulose deposition in primary cell wall	Primary cell wall formation
23	*CKX*	Cytokinin Oxidase	1386	1386	Cytokinin signaling	Hormonal regulators of cambial development
24	*COMT1*	Caffeic acid O- methyl transferase	1101	1966	Catalyzes the conversion of caffeic acid to ferulic acid and of 5-hydroxyferulic acid to sinapic acid	Lignin biosynthesis
25	*CRE*	Cytokinin receptor 1	2994	3392	Cytokinin regulation	Cytokinin signal transduction
26	*DHN*	Dehydrin	513	918	Hydrophilic LEA proteins and accumulate during cellular dehydration	Expressed during dehydration
27	*DIR1*	Dirigent- like protein	498	498	Lignan biosynthesis process	Template for lignin polymerization
28	*DOF1*	Plant specific DNA-binding with one finger domain proteins.	1014	1864	Transcriptional Regulators in plant growth and development	Regulates Interfascicular Cambium Formation and Vascular Tissue Development
29	*DREB1*	Dehydration responsive element binding protein	762	1355	Belong to AP2 TFs and induced during abiotic stress	Stress tolerance
30	*DUF1*	Domain with unknown function	372	372	Unknown	Predicted function in fibre cell wall development
31	*ERF*	Ethylene responsive Transcription factor	681	824	Ethylene signaling	Ethylene signaling
32	*EXPA*	Alpha Expansin	753	1295	Cell wall proteins involved in plant cell growth and developmental processes where cell wall loosening occurs	Plasticize the cellulose-hemicellulose network of primary walls
33	*EXPB*	Beta Expansin	825	1122	Cell wall proteins involved in plant cell growth and developmental processes where cell wall loosening occurs	Cell wall related
34	*F5H*	Ferrulate 5-hydroxylase	1590	2026	Hydroxylation of ferulate to 5-hydroxyferulate	Lignin biosynthesis
35	*FLA1*	Fasciclin like Arabinogalactan protein	945	1281	Diverse developmental roles like differentiation, cell-cell recognition, somatic embryogenesis and PROGRAMMED CELL DEATH	Expressed during xylem differentiation
36	*GA20*	Gibberellin 20-oxidase	1158	1716	Key oxidase enzyme in the biosynthesis of gibberellin	GA signaling
37	*GATA1*	*GATA1* transcription factor	1002	1002	Nitrogen metabolism, blue-light-regulated morphogenesis and circadian rhythm	Unknown
38	*GLU*	Endo glucanase	1506	1938	Catalyzes the hydrolysis of cellulose	Cellulose biosynthesis
39	*GRAS1*	GRAS family TF	1485	1972	Play diverse roles in root and shoot development, gibberellic acid (GA) signaling and phytochrome A signal transduction	Vascular differentiation
40	*GT*	Monoterpeneglucosyltransferase	1383	1467	Monoterpene biosynthesis	Monoterpene biosynthesis
41	*GT 1*	Beta 1–4 xylosyltransferase/Glycosyltransferase	1008	1262	Involved in the synthesis of the hemicellulose glucuronoxylan	Secondary cell wall biogenesis
42	*HB*	Homeodomain TF	951	1774	Plant development, including maintenance of the biosynthesis and signaling pathways of different hormones.	Xylogenesis
43	*HB1 Class III*	Homeodomain Transcription factor Class III	2535	4141	Regulates meristem function	Regulates vascular development
44	*HBI class II*	Homeodomain TF	759	1205	Phototropism and auxin response	Auxin Signaling
45	*HCT*	Hydroxycinnamoyl CoA shikimate	1494	2316	Insertion of the 3-hydroxyl group into monolignol precursors	Lignin biosynthesis
46	*HDKNOX1*	Homeobox Knotted 1-like 7-like TF	930	1434	Repression of progression into specific differentiation steps	Formation and maintenance of shoot apical meristem
47	*HYD*	Predicted alpha/beta hydrolase fold protein	960	1603	Common to several hydrolytic enzymes with diverse functions	Regulate Xylem Cell differentiation
48	*IAA*	IAA binding domain	258	736	Mediators of the auxin signal transduction pathway	IAA signaling
49	*KNOX2*	Class-I KNOTTED1-like homeobox(KNOX) TF	1305	2164	Growth of shoot meristem	Promote meristem function
50	*KOR*	Korrigan/ Endo glucanase	1872	2910	Catalyzes the hydrolysis of cellulose	Cellulose biosynthesis
51	*LAC*	Carbohydrate binding module 48/ Dextrinase	2451	2804	Hydrolysis of starch	Cellulose biosynthesis
52	*LAC2*	Laccase	1659	2255	Oxidative coupling of lignols	Lignin biosynthesis
53	*LBD*	LATERAL ORGAN BOUNDARY domain TF in inflorescences	735	1029	Involved in position of axillary meristem formation	Regulated by Vascular related NAC Domain TFs
54	*LEAFY*	Floricaula / Leafy protein	1080	1080	Floral meristem identity proteins	Express in floral and vegetative meristems
55	*LIM1*	Homeodomain TF	567	1421	Developmental regulators in basic cellular processes such as organizing of cytolskeleton	Lignin biosynthesis
56	*MAN*	1,4 beta Mannanendohydrolase	1308	2052	Depolymerization of these cell wall mannan polysaccharides	Cell wall components/carbohydrate metabolic pathway
57	*MAX*	MORE AXILLARY GROWTH Gene	2094	2659	Regulate auxin transport	Stigolactone related auxin-dependent stimulation of secondary growth
58	*MIBP1*	Metal (copper) ion binding protein	1425	1823	Unknown	Predicted function in xylogenesis
59	*MTS*	Monoterpene synthase	1749	2328	Monoterpene biosynthesis	Monoterpene biosynthesis
60	*MUR3*	XyloglucangalactosyltransferaseExostosin family	1854	2347	Xyloglucan biosynthesis	Xyloglucan biosynthesis
61	*MYB1*	Transcription Factor	768	1629	Second-level master regulators insecondary cell wall biosynthesis	Lignification
62	*MYB2*	Transcription Factor	666	807	Second-level master regulators in secondary cell wall biosynthesis	Lignification
63	*NAM1*	No apical meristem protein	1971	2982	NAC TF involved in development of shoot apical meristem	Vascular differentiation and Signaling
64	*PAAPA*	Hydroxyproline-rich glycoprotein (HRGP) and ‘PAAPA’ motif	519	1189	Probable role in cell wall development	No function assigned
65	*PAE*	Pectin acetyl esterase	1272	2136	Deacetylation of pectin, a major compound of primary cell walls	Pectin biosynthesis
66	*PAL*	Phenyl alanine ammonia lyase	2148	3044	Participates in phenylpropanoid biosynthesis	Lignin biosynthesis
67	*PG*	Polygalacturanase	1530	2288	Degrades polygalacturonan	Cell wall degradation
68	*PIP1*	Aquaporin	864	1268	Membrane intrinsic protein for water channelling	Transport of water and/or small neutral solutes
69	*PL*	PectateLyase	1023	1023	Cleavage of pectate	Pectin biosynthesis
70	*POX1*	Peroxidase	951	1343	Hemoprotein catalyzing the oxidation by hydrogen peroxide	Lignin biosynthesis
71	*PTM5*	MAD box TF	279	279	Flower development	Vascular development
72	*RAB*	Ras-related protein	624	1122	Protein transport. Regulator of membrane traffic from the Golgi apparatus towards the endoplasmic reticulum	Activation of autophagy during wood formation
73	*RNS*	Ribonulcease T2 family	687	856	Hydrolyse RNA	Programmed cell death
74	*ROP1*	RAC- like small GTPase	594	1290	Regulate cellular processes ranging from vesicle trafficking to hormone signaling	Signaling protein during secondary xylem formation
75	*SAMS*	S-adenosylmethionine synthase	1182	1769	Catalyzes the formation of S-adenosylmethionine	Methylation of lignin precursors
76	*SBP1*	Squamosa promoter binding protein TF	1656	2407	Involved in the vegetative to reproductive phase transition; Expression is regulated by MIR156b.	Meristem activation
77	*SCD*	Short chain dehrydrogenase	975	1449	Paticipates in secondary metabolism, stress responses and phytosteroid biosynthesis	Hormone biosynthesis
78	*SND1*	Wood-associated NAC domain transcription factor 1A (WND1A)	1200	1684	Plant developmental process	Key Regulator of secondary wall synthesis in fibres
79	*STM*	Shoot meristemless TF	1128	2348	Meristem formation and maintenance	Regulator of vascular cambium
80	*SuSy1*	Sucrose synthase	2418	2869	Starch and sucrose metabolism	Cellulose biosynthesis
81	*TUA1*	Alpha tubulin	1356	2011	Globular cytoskeleton proteins	Component of microtubules
82	*UBI LIG*	Ubiquitin Ligase	969	1595	Protein ubiquitinization. Targets specific protein substrates for degradation by the proteasome	Programmed cell death
83	*UGDH*	UDP-glucose dehydrogenase	1443	2141	Oxidizes UDP-Glc (UDP-D-glucose) to UDP-GlcA (UDP-D-glucuronate)	Carbohydrate metabolism
84	*UGT*	UDP glucose glucosyltransferase	1410	1687	Catalyze the conjugation of glucose from sugar nucleotides to various substrates	Carbohydrate biosynthesis
85	*UXS1*	UDP-D-Glucuronatecarboxylyase	615	689	Catalyzes the conversion of UDP-d-glucuronate to UDP-d-xylose	Carbohydrate metabolism
86	*VND6*	Vascular-related NAC-domain TF	1047	3267	Master regulator of xylem vessel differentiation	Master regulator of xylem vessel differentiation
87	*VND7*	Vascular-related NAC-domain TF	963	1531	Master regulator of xylem vessel differentiation	Master regulator of xylem vessel differentiation
88	*WND1*	Wood associated NAC TF	1152	1527	Activating the entire secondary wall biosynthetic program	Regulation of secondary wall biosynthesis pathway
89	*WRKY1*	TFs involved in biotic and abiotic stress responses	2256	2870	Plant responses to biotic and abiotic stresses	Cell wall lignification
90	*WUS1*	Homeodomain TF	685	726	A typical Homeodomain TF involved in lateral organ formation and meristem function	Shoot apical meristem formation and maintenance
91	*XCP*	Xylem specific cysteine protease	1131	1564	Cellular autolysis	Programmed cell death
92	*XTH*	Xyloglucanendo-transglycosylase/ hydrolase	894	1223	Cell wall extensibility	Regulates cell growth by strengthening or weakening xyloglucan-cellulose microfibril network
93	*XYL*	Endo 1–4 beta Xylanase	2796	3444	Degrades the linear polysaccharide beta-1,4-xylan into xylose	Carbohydrate active enzymes in secondary cell wall biogenesis. Decreases cellulose crystallinity in cell walls
94	*Znf1*	Zinc finger C3HC4 type (RING)	1113	1542	Cysteine rich domain involved in mediating protein-protein interactions	Ubiquitinization

The formation of the secondary cell wall is driven by the coordinated expression of numerous genes involved in the biosynthesis of cellulose and hemicellulose, lignin, pectin, cell wall proteins and minor soluble and insoluble compounds [54–59], [33, 38–39]. Expressed wood-formation genes show high functional conservation across plant genera and up to 90% of genes expressed in loblolly pine have homologs in *Arabidopsis* [60]. Similarly, a high proportion of poplar ESTs appear to have homologs in the *Arabidopsis* genome [61–62].

The role of transcription factors as master switches in vascular and xylem development has been investigated in detail in poplar, eucalypts, pine and *Arabidopsis*. Highly expressed transcription factors like MYB and NAC families are implicated as critical regulators of vascular differentiation, phenylpropanoid metabolism, xylem differentiation and secondary wall formation. The other important regulators include the homeodomain superfamily of transcription factors (HD-Zip, WOX, KNOX, and ZF-HD), ethylene responsive elements (AP2/ERF domain), bZIP, WRKY and LIM [63–70].

Hormonal regulation of wood formation is well documented and major phyto-hormones playing pivotal role in cambial activity and wood formation include auxin, cytokinin, gibberellic acid, brassinosteroids and ethylene. The receptors of hormone responsive genes and transcription factors are reported to be expressed during cambial development and wood formation [71–74].

The selection of genes in the present study was based on the literature survey as described above and major functional and regulatory genes presumably involved in cambial development and wood formation were selected.

### Validation of Target Enrichment

The array based hybridization enrichment was conducted to capture the 94 xylogenesis-related genes in three species of *Eucalyptus*. The enrichment of the targeted regions after hybridization was validated using the RT-qPCR on pre- and post-capture libraries for target genes *EtCesA1, EtCesA2* and *EtCesA5* and non target genes *EteIF4* and *EtH2B*. The comparison of pre and post hybridization data demonstrated 64 fold, 165 fold and 59 fold enrichments of the target genes, *EtCesA1, EtCesA2* and *EtCesA5* respectively, while no enrichment was observed for the non target genes, *EteIF4* and *EtH2B*.

### Read and Alignment Statistics

The 2 × 100 bp paired end raw reads were subjected to quality checking using SeqQC_V2.2. In *E. camaldulensis* (Ec111), a total of 15.75 million reads were generated and the total number of HQ reads were 13.86 million (88.02%), while in *E. tereticornis* (Et 86), the total number of reads were 17.07 million and the number of HQ reads were 15.14 million (88.69%). In *E. grandis* (Eg9), the total number of reads was 11.41 million with 10.22 million HQ reads (89.59%).

The HQ reads from all the three species were aligned with the *E. grandis* reference sequence using both gapped and un-gapped alignment tools. In *E. camaldulensis*, 170866bp (98.43% read coverage) were aligned with the reference sequence, which had a total sequence length of 173593bp, while in *E. tereticornis*, 170825bp sequence length was aligned with reference with 98.41% coverage. Similarly, in *E. grandis*, 170671bp was aligned with the reference sequence with coverage of 98.32%. The total percent of reference covered with at least 5X depth was 97.71%, 97.86% and 97.12% in *E. camaldulensis, E. tereticornis* and *E. grandis*, respectively, while reference covered with at least 10X read depth was 96.99%, 97.36% and 95.67%, respectively. Similarly, the alignment statistics for reference covered with 20X depth was 95.9%, 96.34% and 93.53% in *E. camaldulensis, E. tereticornis* and *E. grandis*, respectively. The optimized average read depth in *E. camaldulensis* was ∼223X, while in *E. tereticornis* it was calculated as ∼227X. The optimized average read depth in *E. grandis* was ∼199X. The aligned sequence data was deposited in NCBI Short Read Archive with the accession number SRP045253 for *E. tereticornis* (SRX747331), *E. camaldulensis* (SRX669390) and *E. grandis* (SRX747330).

Next generation sequencing platforms produce robust sequence output making high throughput DNA marker discovery feasible and cost effective [75–76]. It was reported that considering all available NGS platforms, Illumina was preferred for *de novo* sequencing, re-sequencing and high-throughput SNP discovery, due to generation of high read depth leading to reference based contig assembly with high confidence [75–77]. The efficiency of this platform in SNP discovery has been well documented in *E. camaldulensis* [78]; *Arabidopsis* [79]; wheat [80–82]; olive [83]; *Solanum* spp. [84]; Douglas—fir [85]; soybean [86–87]; apple [88] and pine [89].

Another important consideration while conducting target enrichment and re-sequencing is the read depth to reliably detect SNPs. It was reported that a minimum of 8X coverage [90] and up to 200X [91] was optimal for SNP calling. In the present study, the read depth was significantly high at ∼223X in *E. camaldulensis*, ∼227X in *E. tereticornis* and ∼199X in *E. grandis*. Similar studies in *Fragaria vesca* documented the average depth as 120X [92], while in *E. camaldulensis*, the average read depth for all the bases was 6124X [78].

Specificity (the number of reads that map to the targeted sequence) is an important aspect of target enrichment experiments. The present study documented high read coverage with *E. camaldulensis* showing 98.43% coverage, *E. tereticornis* with 98.41% coverage and *E. grandis* with coverage of 98.32% with reference sequence, suggesting high specificity of the hybridized probes to the target sequences. Similarly, in an earlier study in *E. camaldulensis*, 94.2% coverage was reported with reference genome of *E. grandis* [78]. In the wheat, NimbleGen array with genomic DNA derived from eight wheat varieties was used for target enrichment and exome sequencing and an average of 38.1% (22%–44.5%) was aligned to the reference sequence [80], while Saintenac and co workers [82] reported an increase in specificity of reads on target to 60% and the number of covered target bases reported was 92%. In *Populus trichocarpa*, an average of 86.8% of base pairs in the bait regions was mapped on the reference sequence [93]. Hence, the high read depth and coverage achieved in the present investigation can be considered optimal for identification of variation with high confidence.

### Identification of Variants (Snvs And Indels) in Three Eucalyptus Species across E. Grandis Reference Genome

The SNVs and InDels present in the sequences aligned with the reference were individually determined for each species. A total of 5905 SNVs were discovered in all three species, which included 2294 SNVs in *E. camaldulensis* (604 and 299 SNVs from gapped and un-gapped alignments, respectively and 1391 SNVs common for both gapped and un-gapped alignments), 2383 SNVs in *E. tereticornis* (636 and 303 SNVs from gapped and un-gapped alignments, respectively and 1444 SNVs common for both alignments), and 1228 SNVs in *E. grandis* (460 and 122 SNVs from gapped and un-gapped alignments, respectively and 646 SNVs common for both alignments) (Table 2).

**Table 2:** SNVs and InDels across 94 genes in three Eucalyptus species.

**S. No.**	**Gene Name**	**Gene ID**	***E. tereticornis***		***E. camaldulensis***		***E. grandis***	
			**SNVs**	**InDels**	**SNVs**	**InDels**	**SNVs**	**InDels**
1	4-coumarate-CoA ligase	*4CL*	41	5	43	2	5	5
2	Aminocyclopropane-1-carboxylate oxidase	*ACO1*	36	6	34	3	30	5
3	Alcohol dehydrogenase	*ADH*	32	7	31	7	26	8
4	APETALA TF	*AP2L*	7	4	6	4	1	4
5	Auxin response factor	*ARF*	26	11	30	12	1	7
6	Auxin response factor	*ARF2*	29	14	22	15	1	12
7	Aspartyl protease	*ASP*	36	0	32	0	33	0
8	S1/P1 nuclease induced during senescence	*BFN1*	25	3	21	3	4	3
9	KNAT knotted like homeobox TF	*BP*	11	7	12	7	2	7
10	Basic region / leucine zipper motif TF	*bZIP*	15	2	13	2	11	2
11	P-coumarate 3-hydroxylation	*C3H*	68	6	68	5	60	5
12	Cinnamate 4-hydroxylase	*C4H*	11	4	12	4	3	1
13	Cinnamyl alcohol dehydrogenase	*CAD1*	14	6	18	5	6	6
14	Coniferaldehyde 5-hydroxylase	*CAld5H*	19	0	19	0	9	0
15	CBF TF	*CCAAT*	2	1	2	2	0	1
16	Caffeoyl-CoA-O -methyltransferase	*CCoAOMT1*	14	9	7	4	4	5
17	Cellulose synthase 1	*CesA1*	22	11	16	10	28	9
18	Cellulose synthase 2	*CesA2*	19	8	29	8	17	6
19	Cellulose synthase 3	*CesA3*	35	11	36	11	27	10
20	Cellulose synthase 4	*CesA4*	51	15	59	15	30	11
21	Cellulose synthase 5	*CesA5*	55	8	62	10	57	8
22	Cellulose synthase 6	*CesA6*	30	13	29	13	25	12
23	Cytokinin oxidase	*CKX*	30	5	27	4	26	5
24	Caffeic acid-O-methyltransferase	*COMT1*	35	7	37	9	26	3
25	Cytokinin receptor 1	*CRE*	31	13	37	14	7	12
26	Dehydrin	*DHN*	20	4	20	3	4	1
27	Dirigent like protein	*DIR1*	2	0	2	1	3	0
28	DNA binding with one finger	*DOF1*	17	0	19	0	4	0
29	Dehydration-Responsive Element-Binding protein	*DREB1*	11	3	16	3	2	2
30	Domain of Unknown function 1	*DUF1*	7	1	13	1	7	1
31	Ethylene responsive transcription factor	*ERF*	19	1	21	1	20	3
32	Alpha expansin	*EXPA*	13	3	17	7	1	3
33	Beta expansin	*EXPB*	29	3	30	2	29	2
34	Ferulate-5-hydroxylase	*F5H*	19	0	17	0	8	0
35	Fasciclin like arabinogalacton	*FLA1*	22	0	17	0	0	1
36	Gibberllin 20-oxidase	*GA20*	12	3	10	4	12	3
37	*GATA1* transcription factor	*GATA1*	7	2	9	0	1	0
38	Endo glucanase	*GLU*	21	4	16	5	16	4
39	GRAS family TF	*GRAS1*	16	3	14	1	4	2
40	Monopterene glycosyl transferases	*GT*	33	2	35	1	14	2
41	Beta 1–4 xylosyltransferase/glycosyl transferase	*GT_1*	15	3	13	3	10	4
42	Homeodomain TF	*HB*	23	10	23	8	11	4
43	Homeodomain TF	*HB1ClassIII*	32	20	22	18	16	17
44	Homeodomain TF	*HBIclassII*	12	3	13	4	3	4
45	Hydroxycinnamoyl CoA shikimate	*HCT*	34	5	37	5	20	1
46	Homeobox knotted 1-like 7-like TF	*HDKNOX1*	9	6	11	5	5	4
47	Predicted alpha/beta hydrolase fold protein	*HYD*	17	6	15	4	7	4
48	IAA binding domain	*IAA*	13	3	9	1	8	1
49	Class-I KNOTTED 1 like homeobox (KNOX)TF	*KNOX2*	22	8	20	8	10	13
50	KORRIGAN /endo glucanase	*KOR*	60	9	54	11	28	11
51	Carbohydrate binding module 48/ dextrinase	*LAC*	26	10	25	13	6	8
52	Laccase	*LAC2*	29	5	23	7	1	5
53	Lateral organ boundary domain TF in infloresceneces	*LBD*	8	5	9	2	0	1
54	Floricaula/ leafy protein	*LEAFY*	31	4	34	3	22	2
55	Homeodomain TF	*LIM1*	17	7	13	6	3	9
56	1,4 beta mannan endohydrolase	*MAN*	15	7	19	6	7	6
57	More axillary growth gene	*MAX*	48	8	56	4	39	2
58	Metal (copper) ion binding	*MIBP1*	55	4	44	2	33	3
59	Monopterene synthase	*MTS*	49	7	44	8	24	4
60	Xyloglucan galactosyl transferase exostosin family	*MUR3*	30	1	28	1	4	1
61	Myeloblastosis TF	*MYB1*	21	5	18	5	2	3
62	Myeloblastosis TF	*MYB2*	10	1	9	3	11	1
63	No apical meristem family protein	*NAM1*	71	7	62	8	8	6
64	Hydroxy proline rich glycoprotein (HRGP)/ PAAPA motif	*PAAPA*	37	3	21	1	6	2
65	Pectin acetyl esterase	*PAE*	26	9	32	10	11	6
66	Phenylalanine ammonia-lyase	*PAL*	75	6	71	4	27	3
67	Poly galacturanase	*PG*	38	7	44	5	8	5
68	Plasma membrane intrinsic protein	*PIP1*	21	3	24	5	12	5
69	Pectate lyase	*PL*	32	1	22	0	20	0
70	Peroxidase	*POX1*	27	3	24	3	12	3
71	MAD box TF	*PTM5*	1	1	1	1	1	1
72	RAS related protein	*RAB*	12	6	11	5	12	6
73	Ribonuclease T2 family	*RNS*	8	3	9	3	13	3
74	RAC like small GTPase	*ROP1*	11	5	8	6	4	4
75	S-Adenosyl methionine synthetase	*SAMS*	37	3	31	3	21	2
76	Squamosapromoter binding protein TF	*SBP1*	33	6	30	6	22	5
77	Sitosterol cello dextrin	*SCD*	17	5	8	7	11	5
78	Wood assocated NAC domain TF 1(WND1)	*SND1*	6	7	5	7	4	6
79	Shoot meristemless TF	*STM*	18	20	17	12	3	7
80	Sucrose synthase	*SuSy1*	85	9	72	9	54	8
81	α-Tubulin	*TUA1*	33	7	21	7	5	4
82	Ubiquitin Ligase	*UBILIG*	9	5	9	4	2	5
83	UDP glucose glucosyl dehydrogenase	*UGDH*	21	6	18	3	12	3
84	UDP glucose glucosyl transferase	*UGT*	40	2	63	1	44	2
85	UDP-D-glucuronate carboxylyase	*UXS1*	9	1	12	0	9	0
86	Vascular related NAC domain TF	*VND6*	22	8	21	5	1	4
87	Vascular related NAC domain TF	*VND7*	9	5	9	4	4	4
88	Wood associated NAC TF	*WND1*	19	2	16	4	5	2
89	TF involved in biotic and abioic stress response	*WRKY1*	32	10	25	9	15	7
90	Homeodomain TF	*WUS1*	3	1	9	1	5	0
91	Xylem-specific papain-like Cysteine Peptidase	*XCP*	21	4	20	3	6	2
92	Xyloglucan transglycosylase	*XTH*	22	2	28	4	12	2
93	Endo 1,4 beta xylanase	*XYL*	48	9	41	8	16	10
94	Zinc finger (C3HC4-type ring finger) TF protein	*Znf1*	22	10	13	6	9	8
**Total**			**2383**	**518**	**2294**	**479**	**1228**	**409**

The presence of SNVs in UTRs and exons were also identified and maximum number of SNVs was recorded in the exon region (4187), while 1226 SNVs were documented in the 3’UTR. A total number of 492 SNVs were identified in the 5’UTR across all the three species (Table 3, 4 & 5). In *E. tereticornis*, the maximum number of SNVs was recorded in *SuSy1* (85), while only one SNV was observed in *PTM5* (S3a Table). In *E. camaldulensis*, a similar trend was observed with maximum of 72 SNVs identified in *SuSy1* and only one SNV recorded in *PTM5* (S4a Table). However, when the *E. grandis* sequences were compared with the reference genome, a maximum of 60 SNVs was observed in *C3H* while a single SNV was documented in several genes, including *AP2L, ARF, ARF2, EXPA, GATA1, LAC2, PTM5, VND6*. No SNVs were detected in *CCAAT, FLA1*, and *LBD* (S5a Table).

**Table 3:** SNV frequency in three Eucalyptus species in 5′UTR region.

		***E. tereticornis***		***E. camaldulensis***		***E. grandis***	
**GENE ID**	**5′UTR_length (bp)**	**No. of SNVs**	**SNV frequency (bp/SNV)**	**No. of SNVs**	**SNV frequency (bp/SNV)**	**No. of SNVs**	**SNV frequency (bp/SNV)**
*4CL*	126	3	42.0	3	42.0	1	126.0
*ACO1*	107	2	53.5	1	107.0	1	107.0
*ADH*	0	na	na	na	na	na	Na
*AP2L*	59	1	59.0	1	59.0	-	
*ARF*	413	4	103.3	3	137.7	1	
*ARF2*	1004	8	125.5	9	111.6	-	
*ASP*	0	na	na	na	na	na	Na
*BFN1*	0	na	na	na	na	na	Na
*BP*	267	-		-		-	
*bZIP*	0	na	na	na	na	na	Na
*C3H*	86	2	43.0	1	86.0	2	43.0
*C4H*	125	3	41.7	1	125.0	-	
*CAD1*	127	2	63.5	5	25.4	-	
*CAld5H*	39	-		-		-	
*CCAAT*	0	na	na	na	na	na	na
*CCoAOMT1*	97	1	97.0	-		-	
*CesA1*	0	na	na	na	na	na	na
*CesA2*	361	-		-		-	
*CesA3*	0	na	na	na	na	na	na
*CesA4*	340	8	42.5	2	170.0	4	85.0
*CesA5*	118	-		1	118.0	1	118.0
*CesA6*	16	-		-		-	
*CKX*	0	na	na	na	na	na	na
*COMT1*	98	1	98.0	-		2	49.0
*CRE*	0	na	na	na	na	na	na
*DHN*	112	4	28.0	1	112.0	1	112.0
*DIR1*	0	na	na	na	na	na	na
*DOF1*	227	1	227.0	1	227.0	-	
*DREB1*	298	5	59.6	8	37.3	-	
*DUF1*	0	na	na	na	na	na	na
*ERF*	0	na	na	na	na	na	na
*EXPA*	43	-		1	43.0	-	
*EXPB*	44	1	44.0	1	44.0	1	44.0
*F5H*	39	-		-		-	
*FLA1*	1	-		-		-	
*GA20*	105	-		-		-	
*GATA1*	0	na	na	na	na	na	na
*GLU*	41	1	41.0	1	41.0	-	
*GRAS1*	213	3	71.0	3	71.0	-	
*GT*	54	3	18.0	3	18.0	-	
*GT 1*	187	-		-		-	
*HB*	305	1	305.0	1	305.0	1	305.0
*HB1 ClassIII*	1090	8	136.3	8	136.3	5	218.0
*HBI class II*	102	-		-		-	
*HCT*	298	4	74.5	9	33.1	3	99.3
*HDKNOX1*	103	-		-		-	
*HYD*	262	2	131.0	1	262.0	2	131.0
*IAA*	91	-		-		-	
*KNOX2*	182	3	60.7	7	26.0	3	60.7
*KOR*	465	9	51.7	9	51.7	7	66.4
*LAC*	0	na	na	na	na	na	na
*LAC2*	52	-		-		-	
*LBD*	35	-		-		-	
*LEAFY*	0	na	na	na	na	na	na
*LIM1*	408	12	34.0	10	40.8	2	204.0
*MAN*	218	-		1	218.0	-	
*MAX*	35	-		-		-	
*MIBP1*	0	na	na	na	na	na	na
*MTS*	230	6	38.3	5	46.0	5	46.0
*MUR3*	493	8	61.6	9	54.8	1	493.0
*MYB1*	429	8	53.6	6	71.5	1	429.0
*MYB2*	79	1	79.0	1	79.0	3	26.3
*NAM1*	307	10	30.7	5	61.4	1	307.0
*PAAPA*	131	-		2	65.5	-	
*PAE*	536	12	44.7	15	35.7	7	76.6
*PAL*	194	6	32.3	5	38.8	-	
*PG*	242	6	40.3	6	40.3	4	60.5
*PIP1*	67	-		-		-	
*PL*	0	na	na	na	na	na	na
*POX1*	42	1	42.0	-		-	
*PTM5*	0	na	na	na	na	na	na
*RAB*	158	2	79.0	2	79.0	1	158.0
*RNS*	15	-		-		-	
*ROP1*	241	-		-		-	
*SAMS*	271	13	20.8	9	30.1	2	135.5
*SBP1*	451	4	112.8	2	225.5	1	451.0
*SCD*	181	6	30.2	1	181.0	1	181.0
*SND1*	218	2	109.0	1	218.0	3	72.7
*STM*	669	5	133.8	4	167.3	2	334.5
*SuSy1*	140	2	70.0	1	140.0	-	
*TUA1*	318	11	28.9	9	35.3	-	
*UBILIG*	434	6	72.3	4	108.5	1	434.0
*UGDH*	258	2	129.0	2	129.0	-	
*UGT*	11	-		-		-	
*UXS1*	0	na	na	na	na	na	na
*VND6*	422	3	140.7	4	105.5	-	
*VND7*	117	1	117.0	1	117.0	-	
*WND1*	77	-		-		-	
*WRKY1*	390	8	48.8	5	78.0	-	
*WUS1*	41	-		-		-	
*XCP*	30	-		-		-	
*XTH*	119	1	119.0	-		1	119.0
*XYL*	362	6	60.3	3	120.7	2	181.0
*Znf1*	180	1	180.0	1	180.0	1	180.0
**Total**	**16246**	**223**	78.49*	**195**	101.11*	**74**	170.42*

**na: Not applicable**

* **denotes average SNV frequency**

**Table 4:** SNV frequency in three Eucalyptus species in Exon region.

		***E. tereticornis***		***E. camaldulensis***		***E. grandis***	
**GENE ID**	**Exon length (bp)**	**No. of SNVs**	**SNV frequency (bp/SNV)**	**No. of SNVs**	**SNV frequency (bp/SNV)**	**No. of SNVs**	**SNV frequency (bp/SNV)**
*4CL*	1635	37	44.2	38	43.0	4	408.8
*ACO1*	963	28	34.4	29	33.2	26	37.0
*ADH*	1896	18	105.3	23	82.4	20	94.8
*AP2L*	738	2	369.0	2	369.0	-	
*ARF*	2364	19	124.4	22	107.5	-	
*ARF2*	2520	17	148.2	12	210.0	1	2520.0
*ASP*	1218	36	33.8	32	38.1	33	36.9
*BFN1*	912	16	57.0	10	91.2	3	304.0
*BP*	1164	7	166.3	10	116.4	2	582.0
*bZIP*	858	15	57.2	13	66.0	11	78.0
*C3H*	1530	55	27.8	50	30.6	51	30.0
*C4H*	1518	6	253.0	8	189.8	3	506.0
*CAD1*	1158	12	96.5	13	89.1	6	193.0
*CAld5H*	1590	17	93.5	17	93.5	9	176.7
*CCAAT*	453	2	226.5	2	226.5	-	
*CCoAOMT1*	741	5	148.2	4	185.3	-	
*CesA1*	2937	22	133.5	16	183.6	28	104.9
*CesA2*	2775	19	146.1	29	95.7	17	163.2
*CesA3*	3126	35	89.3	36	86.8	27	115.8
*CesA4*	3243	38	85.3	50	64.9	21	154.4
*CesA5*	1710	45	38.0	48	35.6	47	36.4
*CesA6*	2691	29	92.8	28	96.1	23	117.0
*CKX*	1386	30	46.2	27	51.3	26	53.3
*COMT1*	1101	28	39.3	28	39.3	23	47.9
*CRE*	2994	27	110.9	33	90.7	5	598.8
*DHN*	513	10	51.3	11	46.6	2	256.5
*DIR1*	498	2	249.0	2	249.0	3	166.0
*DOF1*	1014	10	101.4	11	92.2	4	253.5
*DREB1*	762	4	190.5	7	108.9	-	
*DUF1*	372	7	53.1	13	28.6	7	53.1
*ERF*	681	18	37.8	19	35.8	19	35.8
*EXPA*	753	7	107.6	8	94.1	-	
*EXPB*	825	19	43.4	19	43.4	23	35.9
*F5H*	1590	17	93.5	15	106.0	8	198.8
*FLA1*	945	20	47.3	14	67.5	-	
*GA20*	1158	7	165.4	6	193.0	7	165.4
*GATA1*	1002	7	143.1	9	111.3	1	1002.0
*GLU*	1506	16	94.1	11	136.9	14	107.6
*GRAS1*	1485	11	135.0	6	247.5	4	371.3
*GT*	1383	29	47.7	31	44.6	14	98.8
*GT 1*	1008	14	72.0	12	84.0	9	112.0
*HB*	951	13	73.2	12	79.3	4	237.8
*HB1 ClassIII*	2535	15	169.0	9	281.7	7	362.1
*HBI class II*	759	7	108.4	7	108.4	3	253.0
*HCT*	1494	21	71.1	25	59.8	14	106.7
*HDKNOX1*	930	5	186.0	4	232.5	3	310.0
*HYD*	960	6	160.0	6	160.0	3	320.0
*IAA*	258	4	64.5	4	64.5	5	51.6
*KNOX2*	1305	5	261.0	3	435.0	1	1305.0
*KOR*	1872	40	46.8	31	60.4	6	312.0
*LAC*	2451	14	175.1	15	163.4	6	408.5
*LAC2*	1659	19	87.3	16	103.7	-	
*LBD*	735	6	122.5	7	105.0	-	
*LEAFY*	1080	31	34.8	34	31.8	22	49.1
*LIM1*	567	1	567.0	2	283.5	1	567.0
*MAN*	1308	6	218.0	9	145.3	5	261.6
*MAX*	2094	40	52.4	45	46.5	34	61.6
*MIBP1*	1425	40	35.6	31	46.0	28	50.9
*MTS*	1749	34	51.4	30	58.3	13	134.5
*MUR3*	1854	22	84.3	19	97.6	3	618.0
*MYB1*	768	7	109.7	5	153.6	1	768.0
*MYB2*	666	9	74.0	8	83.3	8	83.3
*NAM1*	1971	46	42.8	43	45.8	6	328.5
*PAAPA*	519	17	30.5	12	43.3	6	86.5
*PAE*	1272	9	141.3	10	127.2	4	318.0
*PAL*	2148	43	50.0	47	45.7	19	113.1
*PG*	1530	14	109.3	17	90.0	3	510.0
*PIP1*	864	14	61.7	15	57.6	7	123.4
*PL*	1023	32	32.0	22	46.5	20	51.2
*POX1*	951	16	59.4	16	59.4	9	105.7
*PTM5*	279	1	279.0	1	279.0	1	279.0
*RAB*	624	4	156.0	3	208.0	4	156.0
*RNS*	687	5	137.4	5	137.4	7	98.1
*ROP1*	594	3	198.0	3	198.0	1	594.0
*SAMS*	1182	17	69.5	16	73.9	13	90.9
*SBP1*	1656	23	72.0	23	72.0	12	138.0
*SCD*	975	6	162.5	4	243.8	8	121.9
*SND1*	1200	3	400.0	3	400.0	1	1200.0
*STM*	1128	6	188.0	2	564.0	-	
*SuSy1*	2418	72	33.6	68	35.6	49	49.3
*TUA1*	1356	18	75.3	8	169.5	1	1356.0
*UBILIG*	969	1	969.0	3	323.0	1	969.0
*UGDH*	1443	12	120.3	11	131.2	2	721.5
*UGT*	1410	35	40.3	57	24.7	40	35.3
*UXS1*	615	7	87.9	10	61.5	7	87.9
*VND6*	1047	6	174.5	3	349.0	1	1047.0
*VND7*	963	6	160.5	7	137.6	4	240.8
*WND1*	1152	15	76.8	11	104.7	3	384.0
*WRKY1*	2256	23	98.1	19	118.7	14	161.1
*WUS1*	685	3	228.3	9	76.1	5	137.0
*XCP*	1131	6	188.5	7	161.6	4	282.8
*XTH*	894	16	55.9	22	40.6	10	89.4
*XYL*	2796	13	215.1	33	84.7	11	254.2
*Znf1*	1113	21	53.0	12	92.8	7	159.0
**Total**	**124987**	**1621**	126.78*	**1618**	125.61*	**948**	306.72*

**na: Not applicable**

* **denotes average SNV frequency**

**Table 5:** SNV frequency in three Eucalyptus species in 3′ UTR region.

		***E. tereticornis***		***E. camaldulensis***		***E. grandis***	
**GENE ID**	**3′UTR_length (bp)**	**No. of SNVs**	**SNV frequency (bp/SNV)**	**No. of SNVs**	**SNV frequency (bp/SNV)**	**No. of SNVs**	**SNV frequency (bp/SNV)**
*4CL*	307	1	307.0	2	153.5	-	
*ACO1*	226	6	37.7	4	56.5	3	75.3
*ADH*	277	14	19.8	8	34.6	6	46.2
*AP2L*	255	4	63.8	3	85.0	1	255.0
*ARF*	451	3	150.3	5	90.2	-	
*ARF2*	425	4	106.3	1	425.0	-	
*ASP*	0	na	na	na	na	na	na
*BFN1*	293	9	32.6	11	26.6	1	293.0
*BP*	389	4	97.3	2	194.5	-	
*bZIP*	0	na	na	na	na	na	na
*C3H*	432	11	39.3	17	25.4	7	61.7
*C4H*	285	2	142.5	3	95.0	-	
*CAD1*	298	-		-		-	
*CAld5H*	397	2	198.5	2	198.5	-	
*CCAAT*	0	na	na	na	na	na	na
*CCoAOMT1*	428	8	53.5	3	142.7	4	107.0
*CesA1*	0	na	na	na	na	na	na
*CesA2*	0	na	na	na	na	na	na
*CesA3*	0	na	na	na	na	na	na
*CesA4*	368	5	73.6	7	52.6	5	73.6
*CesA5*	308	10	30.8	13	23.7	9	34.2
*CesA6*	501	1	501.0	1	501.0	2	250.5
*CKX*	0	na	na	na	na	na	na
*COMT1*	767	6	127.8	9	85.2	1	767.0
*CRE*	398	4	99.5	4	99.5	2	199.0
*DHN*	293	6	48.8	8	36.6	1	293.0
*DIR1*	0	na	na	na	na	na	na
*DOF1*	623	6	103.8	7	89.0	-	
*DREB1*	295	2	147.5	1	295.0	2	147.5
*DUF1*	0	na	na	na	na	na	na
*ERF*	143	1	143.0	2	71.5	1	143.0
*EXPA*	499	6	83.2	8	62.4	1	499.0
*EXPB*	253	9	28.1	10	25.3	5	50.6
*F5H*	397	2	198.5	2	198.5	-	
*FLA1*	335	2	167.5	3	111.7	-	
*GA20*	453	5	90.6	4	113.3	5	90.6
*GATA1*	0	na	na	na	na	na	na
*GLU*	391	4	97.8	4	97.8	2	195.5
*GRAS1*	274	2	137.0	5	54.8	-	
*GT*	30	1	30.0	1	30.0	-	
*GT 1*	67	1	67.0	1	67.0	1	67.0
*HB*	518	9	57.6	10	51.8	6	86.3
*HB1 ClassIII*	516	9	57.3	5	103.2	4	129.0
*HBI class II*	344	5	68.8	6	57.3	-	
*HCT*	524	9	58.2	3	174.7	3	174.7
*HDKNOX1*	401	4	100.3	7	57.3	2	200.5
*HYD*	381	9	42.3	8	47.6	2	190.5
*IAA*	387	9	43.0	5	77.4	3	129.0
*KNOX2*	677	14	48.4	10	67.7	6	112.8
*KOR*	573	11	52.1	14	40.9	15	38.2
*LAC*	353	12	29.4	10	35.3	-	
*LAC2*	544	10	54.4	7	77.7	1	544.0
*LBD*	259	2	129.5	2	129.5	-	
*LEAFY*	0	na	na	na	na	na	na
*LIM1*	446	4	111.5	1	446.0	-	
*MAN*	526	9	58.4	9	58.4	2	263.0
*MAX*	530	8	66.3	11	48.2	5	106.0
*MIBP1*	398	15	26.5	13	30.6	5	79.6
*MTS*	349	9	38.8	9	38.8	6	58.2
*MUR3*	0	na	na	na	na	na	na
*MYB1*	432	6	72.0	7	61.7	-	
*MYB2*	62	-		-		-	
*NAM1*	704	15	46.9	14	50.3	1	704.0
*PAAPA*	539	20	27.0	7	77.0	-	
*PAE*	328	5	65.6	7	46.9	-	
*PAL*	702	26	27.0	19	36.9	8	87.8
*PG*	516	18	28.7	21	24.6	1	516.0
*PIP1*	337	7	48.1	9	37.4	5	67.4
*PL*	0	na	na	na	na	na	na
*POX1*	350	10	35.0	8	43.8	3	116.7
*PTM5*	0	na	na	na	na	na	na
*RAB*	340	6	56.7	6	56.7	7	48.6
*RNS*	154	3	51.3	4	38.5	6	25.7
*ROP1*	455	8	56.9	5	91.0	3	151.7
*SAMS*	316	7	45.1	6	52.7	6	52.7
*SBP1*	300	6	50.0	5	60.0	9	33.3
*SCD*	293	5	58.6	3	97.7	2	146.5
*SND1*	266	1	266.0	1	266.0	-	
*STM*	551	7	78.7	11	50.1	1	551.0
*SuSy1*	311	11	28.3	3	103.7	5	62.2
*TUA1*	337	4	84.3	4	84.3	4	84.3
*UBILIG*	192	2	96.0	2	96.0	-	
*UGDH*	440	7	62.9	5	88.0	10	44.0
*UGT*	266	5	53.2	6	44.3	4	66.5
*UXS1*	74	2	37.0	2	37.0	2	37.0
*VND6*	1798	13	138.3	14	128.4	-	
*VND7*	451	2	225.5	1	451.0	-	
*WND1*	298	4	74.5	5	59.6	2	149.0
*WRKY1*	224	1	224.0	1	224.0	1	224.0
*WUS1*	0	na	na	na	na	na	na
*XCP*	403	15	26.9	13	31.0	2	201.5
*XTH*	210	5	42.0	6	35.0	1	210.0
*XYL*	286	29	9.9	5	57.2	3	95.3
*Znf1*	249	-		-		1	249.0
**Total**	**30768**	**539**	86.61*	**481**	100.23*	**206**	176.08*

**na: Not applicable**

* **denotes average SNV frequency**

The SNV frequency was calculated for exon and the UTR regions individually in each species. The SNV frequency in 5′UTR of *E. tereticornis, E. camaldulensis* and *E. grandis* was 1/78.49bp, 1/101.11bp and 1/170.42 respectively, while SNV frequency in the exon region was 1/126.78, 1/125.61 and 1/306.72 for *E. tereticornis, E. camaldulensis* and *E. grandis* respectively. In 3′UTR, the SNV frequency was 1/86.61, 1/100.23 and 1/176.08 for *E. tereticornis, E. camaldulensis* and *E. grandis* respectively (Table 3, 4 & 5).

Further, the presence of SNVs in pair-wise combination between the three *Eucalyptus* species was also conducted. The gene-wise presence of ambiguous nucleotides was not considered and SNV with no ambiguity was mapped on the candidate genes (S6 Table). When *E. camaldulensis* and *E. tereticornis* were compared, a total of 317 SNVs were documented with a minimum of one SNV in *4CL, bZIP, CCoAOMT1, CesA3, EXPA, GRAS1, NAM1, PIP1, PTM5, SBP1, SND1, STM, SuSy1, TUA1, VND7* and a maximum of 25 SNVs in *LAC*. Larger number of SNVs were recorded when *E. grandis* was compared with *E. tereticornis* and *E. camaldulensis* with 875 and 1014 SNVs respectively. In both pair-wise combinations, the maximum number of SNVs was observed in *LAC* with 53 SNVs when compared across *E. camaldulensis* and 46 SNVs when compared across *E. tereticornis*.

The presence of InDels were also detected when the sequences of 94 genes were compared individually across the reference and a total of 1406 InDels were discovered with the size range of 1–24 nucleotides (Table 2). The position of InDels in exons and UTRs was also determined and the total number documented was 843, 309 and 254 in exons, 3’UTR and 5’UTR, respectively (Table 6). In *E. tereticornis*, a total of 518 InDels were detected and a maximum of 20 InDels was recorded in the transcription factor *HB1 Class III*, while a single InDel was documented in several genes including *CCAAT, DUF1,ERF, MUR3,MYB2,PL, PTM5,UXS1* and *WUS1*. No InDels were recorded in *ASP, CAld5H, DOF1, F5H, DIR1*, and *FLA1* (S3b Table). In *E. camaldulensis*, a total of 479 InDels were recorded and the maximum number of InDels was discovered in *HB1ClassIII* (18), while only a single InDel was identified in *DIR1, DUF1, ERF, GRAS1, GT, IAA, MUR3, PAAPA, PTM5, UGT* and *WUS1*. InDels were not detected in *ASP, CAld5H, DOF1, F5H, FLA1, GATA1, PL* and *UXS1* (S4b Table). In *E. grandis*, a total of 409 InDels were discovered and a maximum of 17 InDels was documented in *HB1ClassIII*, while only a single InDel was identified in *FLA1, DUF1, IAA, MUR3, PTM5, CCAAT, LBD, DHN, MYB2, C4H* and *HCT*. InDels were not found in *ASP, CAld5H, DOF1, F5H, GATA1, PL, UXS1, DIR1* and *WUS1* (S5b Table). The InDel frequency was calculated for each species (Table 6). The InDel frequency (bp/InDel) was the highest in the exon region for all the three species with 411.14, 446.38 and 482.58 in *E. tereticornis, E. camaldulensis* and *E. grandis*, respectively. The total InDel frequency was 332.05, 359.08 and 420.54 bp per InDel in *E. tereticornis, E. camaldulensis* and *E. grandis* respectively, across the all the genes selected (Table 6).

**Table 6:** InDel frequency in three Eucalyptus species.

**Region**	**No. of InDels**	**Length(bp)**	**InDel frequency(bp/InDel)**
***E. tereticornis***			
5′UTR	99	16246	164.10
EXON	304	124987	411.14
3′UTR	115	30768	267.55
**Total**	**518**	**172001**	332.05*
***E. camaldulensis***			
5′UTR	89	16246	182.54
EXON	280	124987	446.38
3′UTR	110	30768	279.71
**Total**	**479**	**172001**	359.08*
***E. grandis***			
5′UTR	66	16246	246.15
EXON	259	124987	482.58
3′UTR	84	30768	366.29
**Total**	**409**	**172001**	420.54*

* **denotes average InDel frequency**

Similarly, the presence of InDels was also documented in pair-wise combination and a total of 731 and 699 InDels were detected across *E. grandis* & *E. tereticornis* and *E. grandis* & *E. camaldulensis*, respectively. A total of 702 InDels were detected between *E. camaldulensis* and *E. tereticornis*. Maximum number of InDels across all combinations was observed in *HB1 Class III* transcription factor with 26 InDels when compared between *E. grandis* and *E. tereticornis*, 27 InDels between *E. grandis* and *E. camaldulensis* and 27 InDels between *E. camaldulensis* and *E. tereticornis*. A minimum of one InDel was documented across several genes like *FLA1; DIR1, EXPB, FLA1, WUS1* and *DIR1, DUF1, PL, UXS1* in *E. grandis* & *E. tereticornis*; *E. grandis* & *E. camaldulensis* and *E. camaldulensis* & *E. tereticornis* respectively (S7a,b,c Table).

The abundance of SNPs / SNVs in plant genome and the availability of cost effective technologies for genotyping has made high-throughput SNP genotyping pivotal for genetic mapping, gene discovery, germplasm characterization and population genomics [94]. NGS based SNP discovery is reported in several crop like wheat [80], [81], [82]; *Eucalyptus* [95]; rice [96]; barley [97]; cotton [98]; soybean [86]; potato [99]; *Arabidopsis* [100]; maize [101] and several other species. Use of SNP marker panels for genetic analysis has been widely explored in less domesticated crop [102] and trees [103–105]. SNP genotyping in Eucalypts species is reported from *E. grandis* [35], *E. globulus, E. nitens, E. camaldulensis* and *E. loxophleba* [16], inter-specific hybrids of *Eucalyptus* [106], *E. pilularis* [107], *E. globulus* [108] and *E. camaldulensis* [41,78].

The SNP frequency in *Eucalyptus* species is considered to be one of the highest in woody species due to its recent domestication, large population size and outbred mating system [94]. Kulheim and coworkers [16] reported that the SNP density in *E. nitens* was 1/33bp, 1/31 bp in *E. globulus*, while in *E. camaldulensis* and *E. loxophleba* it was significantly high at 1/16bp and 1/17bp respectively. However, a later study showed that the SNP frequency was 1/83.9bp in *E. camaldulensis* [78]. In the present study, the SNV frequency ranged from 1/78.49bp to 1/306.72bp across different genic regions of *E. camaldulensis, E. tereticornis* and *E. grandis*. Recently, the SNP frequency in inter-specific hybrids of Eucalypts was documented as 1/133bp [109], suggesting that the SNP frequency was depended on the target region. In heterozygous species, the SNP frequency is generally high as documented in pine with 1/102.6bp [110], grapevine with 1/64bp [111], maize with 1/60bp [112] and rye which registered a SNP frequency of 1 SNP at 52bp interval [113].

Insertion and deletion polymorphisms (InDels) are an important source of genomic variation in plant and animal genomes. Mechanisms such as insertion and excision of transposable elements, slippage in simple sequence replication, errors in DNA synthesis and repair, recombination and unequal crossover can result in the formation of InDels [114–115]. However, accurate genotyping from low-coverage sequence data can be challenging [116]. Further, polymorphism in short InDels is increasingly being used as an important marker in humans [117], *Drosophila melanogaster* [118] and *G. gallus* [119]. Report on InDel genotyping in plants are limited to rice [120], *Arabidopsis thaliana* [121], *Citrus clementina* [122] and *Phaseolus vulgaris* [123]. In tree species, InDel discovery is reported from *Salix* spp. [124] and *Populus* spp. [125–126]. InDel markers for species discrimination have been reported in *E. grandis* and *E. gunnii* [39] and *Populus* spp. [125,127].

In the present study, high number InDels in the size range of 1–24 nucleotides were documented in the three *Eucalypts* species at a frequency of 332.05, 359.08 and 420.54 bp per InDel in *E. tereticornis, E. camaldulensis* and *E. grandis*, respectively. This is higher than the earlier reported InDel frequency of 1.5 InDel/1000 bp [115] in *Eucalyptus* genome and 1/2756bp in inter-specific hybrid population [109]. Similarly, in *Pinus taeda*, Kong *et al*. [128] reported that InDels were infrequent with only 0.67% frequency in targeted regions. The probable reason for this variance in the present investigation could be due to the highly divergent genotypes selected in the present study, indicating that InDels could be a useful marker for genetic analysis in *Eucalyptus* species.

## Conclusion

The NGS platforms have brought in paradigm shift in understanding the different aspects of plant biology especially in model species and plants with small genome. Its downstream usefulness in linkage map construction, genetic diversity analyses, association mapping, and marker—assisted selection has been demonstrated in several plants [129]. However, sequencing of complete genomes cannot be regularly employed due to high cost and computational limitations in handling robust informatics data. With availability of complexity reduction strategies, sequencing of sub-genomic regions by on-array/in-solution target enrichment technology has provided an efficient alternate strategy to amplicon re-sequencing for SNP/ SNV discovery [130]. In the present study, this strategy was implemented in re-sequencing ninety four genes across three Eucalypts species. This study has also revealed that target enrichment strategy can be successfully used for identification of markers (SNVs and InDels) for future use in QTL and association mapping studies in *Eucalyptus* species.

## Supporting Information

S1 Table Primer pairs used for RT-qPCR to confirm enrichment of targeted genes. (DOC)

S2 Table Functional Annotation of selected genes across *E. grandis* genome sequence using Phytozome v10. (XLSX)

S3 Table A, Details of SNVs documented in *E. tereticornis* across reference sequence. B, Details of InDels documented in *E. tereticornis* across reference sequence.
(XLS)

S4 Table A, Details of SNVs documented in *E. camaldulensis* across reference sequence. B, Details of InDels documented in *E. camaldulensis* across reference sequence.
(XLS)

S5 Table A, Details of SNVs documented in *E. grandis* across reference sequence. B, Details of InDels documented in *E. grandis* across reference sequence.
(XLS)

S6 Table Presence of SNVs in Pair-wise comparison across three *Eucalyptus* species. (XLS)

S7 Table A, Presence of InDels in Pair-wise comparison across *E. grandis* and *E. tereticornis*. B, Presence of InDels in Pair-wise comparison across *E. grandis* and *E. camaldulensis*. C, Presence of InDels in Pair-wise comparison across *E. camaldulensis* and *E. tereticornis*.
(XLSX)
