# Supplementary material for: Development of Genetic Markers in Eucalyptus Species by Target Enrichment and Exome Sequencing
Source: PLoS One. 2015 Jan 20;10(1):e0116528. doi: 10.1371/journal.pone.0116528 (PMC4300219; doi:10.1371/journal.pone.0116528)
Supplement: S1 Table — (DOC) [file pone.0116528.s001.doc]

**Table S1** Primer pairs used for RT-qPCR to confirm enrichment of targeted genes

| **Primer pairs for targeted genes** | | | |
| --- | --- | --- | --- |
| **Primer ID** | **Gene name** | **Gene ID** | **Sequence (5’🡪3’)** |
| CesA2_FP_681 | Cellulose synthase 2 | *EtCesA2* | CCTGCGCCTCATAGTCCTAG |
| CesA2_RP_831 |  |  | TGTCGGGTTCCACTTAGGG |
|  | | | |
| CesA1_FP_2140 | Cellulose synthase 1 | *EtCesA1* | CACCAAGTTCTACGATGGGC |
| CesA1_RP_2290 |  |  | GAGGGAGGGATGTGAATGG |
|  | | | |
| CesA5_FP_1681 | Cellulose synthase 5 | *EtCesA5* | GGGTCGGCAGAATCGTACTC |
| CesA5_RP_1831 |  |  | CAATTAGCAGTTGATGCCGC |

| **Primer pairs for non-targeted genes** | | | |
| --- | --- | --- | --- |
| **Primer ID** | **Gene name** | **Gene ID** | **Sequence (5’🡪3’)** |
| eIF4B_FP | Eukaryotic initiation factor | *EteIF4* | CCCAAATATGAACCGTCCA |
| eIF4B_RP |  |  | GTTCGATCCATAGCGTCC |
|  | | | |
| H2B_FP | Histone protein | *EtH2B* | GAAGAAGCGGGTGAAGAAGA |
| H2B_RP |  |  | GGCGAGTTTCTCGAAGATGT |
